# Supplementary figures and images for: Modelling organophosphate intoxication in C. elegans highlights nicotinic acetylcholine receptor determinants that mitigate poisoning
Source: PLoS One. 2023 Apr 21;18(4):e0284786. doi: 10.1371/journal.pone.0284786 (PMC10121051; doi:10.1371/journal.pone.0284786)

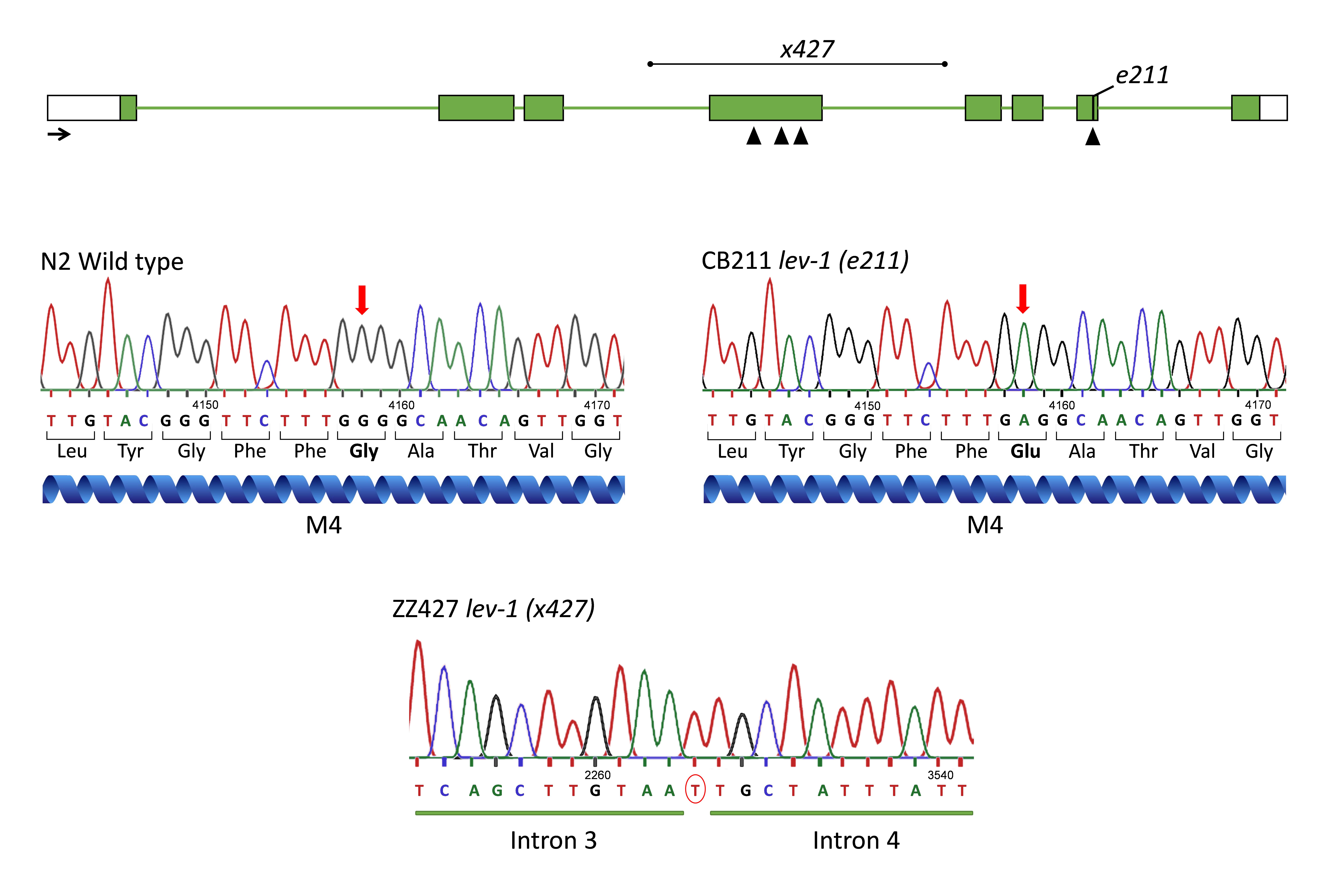

Supplement: S1 Fig — Genomic organization of lev-1 locus indicating the position of the single point mutation in e211 and the deletion in x427 alleles. CB211 lev-1 (e211) mutant strain contained a G to A missense mutation identified in exon 7 of the genomic DNA. This leads to a glycine to glutamic acid exchange at the fourth transmembrane domain (M4). The strain ZZ427 lev-1 (x427) contains a deletion of 1,267 bp from intron 3 to intron 4 and a T insertion. This causes a LEV-1 protein lacking the first, second and third transmembrane domain. Black arrow represents 100 bp and the sense of transcription. Black triangles in the genomic DNA represents the position of the four transmembrane domains. Chromatograms corresponds to the 5’ to 3’ readout of the minus strand. The position indicated in each chromatogram corresponds to the position of the respective base from the ATG starting codon in the genomic DNA of N2 wild type. (JPG) [file pone.0284786.s001.jpg]
